# *Salvia miltiorrhiza* for Viral Myocarditis: Multi-Computational Pharmacological Exploration and Meta-Analytic Efficacy Validation

**DOI:** 10.3390/ijms262311753

**Published:** 2025-12-04

**Authors:** Xingxin Cao, Mingxue Li, Xueqian Xie, Zhun Feng, Weihua Jin, Yanyan Li, Fengmei Yang, Suqin Duan, Zhanlong He

**Affiliations:** 1 Institute of Medical Biology, Chinese Academy of Medical Sciences & Peking Union Medical College, Kunming 650118, China; caoxingxin@student.pumc.edu.cn (X.C.);; 2 College of Life Sciences, Yunnan University, Kunming 650500, China

**Keywords:** viral myocarditis, *salvia miltiorrhiza*, network pharmacology, machine learning, in silico docking, molecular dynamic simulation, meta-analysis

## Abstract

Viral myocarditis (VMC) is the predominant type of myocarditis and currently lacks specific therapies. *Salvia miltiorrhiza* (Danshen) injection has demonstrated beneficial effects as a supplementary VMC treatment, yet its pharmacological mechanisms are ambiguous, and its efficacy lacks robust evidence. This study aims to preliminarily address these issues through computational approaches and meta-analysis. Using network pharmacology, we identified 257 therapeutic targets, 106 hub genes, and 4 key *S. miltiorrhiza* ingredients implicated in VMC treatment. Integrating transcriptome data with LASSO and SVM machine learning algorithm yielded six core therapeutic targets from the hub genes—*TNF*, *JUN*, *PECAM1*, *KDR*, *TIMP1*, and *EPAS1*—which are primarily associated with anti-inflammatory activity, vascular remodeling, and fibrosis suppression. GO analysis identified the “inflammatory response” as the most prominent biological process. Concurrently, the PI3K-Akt, TNF, and HIF-1 signaling pathways—each closely associated with inflammation—appeared among the top 20 KEGG pathways. Overall, these results indicate that suppressing excessive inflammation is likely the primary pharmacological mechanism. In molecular docking, four key ingredients—dan-shexinkum D, danshenol A, cryptotanshinone, and methylrosmarinate—exhibited strong binding to the core therapeutic targets, with dan-shexinkum D showing the lowest total binding energy and stable binding confirmed by molecular dynamics simulations. The meta-analysis indicates that *S. miltiorrhiza* injection improves clinical outcomes and significantly reduces TNF-α, hs-CRP, CK-MB, cTnT, and H-FABP levels. This study used multiple computational approaches to explore the pharmacological mechanisms and identify key active components of *S. miltiorrhiza* in treating VMC, thereby establishing an evidence-based foundation and providing preliminary groundwork for subsequent clinical application and translational research.

## 1. Introduction

Myocarditis, characterized by localized or diffuse inflammatory lesions in the myocardium of diverse etiology, is reported to have an approximate 4% all-cause mortality rate among hospitalized acute cases in the UK [1,2]. A retrospective analysis spanning 16 years and involving 684 myocarditis patients across 19 hospitals revealed that about 25% of these patients experienced symptoms like left ventricular systolic dysfunction, ventricular arrhythmias, or acute heart failure [3]. Mammalian cardiomyocytes are essentially nonrenewable after death, so the cell loss caused by myocarditis can precipitate acute heart failure, sudden cardiac death, or chronic dilated cardiomyopathy, posing a major threat to human health [4,5]. Viral infections often result in the abnormal death of cardiomyocytes and are the leading cause of myocarditis, referred to as viral myocarditis (VMC), which has an estimated incidence of 10 to 22 cases per 100,000 individuals [6,7,8]. A variety of viruses can induce VMC, including coxsackievirus, echovirus, adenovirus, human immunodeficiency virus, influenza virus, coronavirus, parvovirus B19, and human herpesvirus 6 [9,10]. Because no specific therapies exist for VMC, clinical management relies on combined symptomatic treatments after assessing acute severity and clinical presentation, so novel therapeutic strategies urgently require investigation and synthesis [11,12].

Traditional Chinese medicine (TCM), notably Chinese medicine injections (CHIs) derived from herbal sources, is widely used as an adjuvant in combination regimens for VMC, and *Salvia miltiorrhiza* (Danshen) injection is considered one of the most effective CHIs [13,14]. Previous studies indicate that *S. miltiorrhiza* and its constituents exert diverse pharmacological effects, including modulation of immune function, protection against oxidative DNA damage, regulation of inflammatory responses, antibacterial activity and inhibition of viral proliferation [15,16,17]. Despite the widespread clinical use of *S. miltiorrhiza* and its injection as an adjunct therapy, its pharmacological mechanisms and key bioactive components in VMC remain insufficiently characterized, and no rigorous systematic review or meta-analysis evaluating efficacy has yet validated its efficacy. These gaps limit the translational application of *S. miltiorrhiza* and the clinical use of its injection for VMC. The exploration of pharmacological mechanisms and the identification of key components can further inform drug development. For example, a mixed-ingredient injection derived from *S. miltiorrhiza* (Salvianolic acid injection) has been launched in China and is now widely used clinically to treat cerebral infarction, coronary heart disease, and related conditions, where it has demonstrated favorable efficacy [18,19]. Therefore, this study aims to preliminarily elucidate the molecular mechanisms—identifying core therapeutic targets, principal pharmacological effects, and key active ingredients—by applying computational methods, including network pharmacology, machine learning, in silico docking, and molecular dynamics simulation, to determine the core elements of the treatment process. Meanwhile, relevant literature will be retrieved to establish a meta-analysis on the efficacy of *S. miltiorrhiza* injection, aiming to provide evidence to guide its adjunctive use in VMC.

## 2. Results

### 2.1. Network Pharmacology Analysis of Salvia miltiorrhiza Against Viral Myocarditis

**Figure 1:**
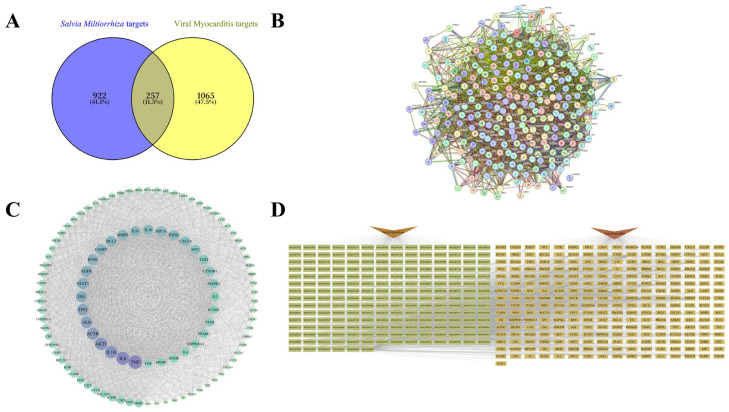
Network pharmacology analysis of *Salvia miltiorrhiza* for viral myocarditis. (**A**) Therapeutic targets were determined by the intersection of targets affected by *S. miltiorrhiza* and those associated with VMC. (**B**) Protein–protein Interaction (PPI) network of the therapeutic targets. (**C**) Network of hub genes identified using Degree, Betweenness, and Closeness metrics in Cytoscape 3.10.3. (**D**) “*Salvia miltiorrhiza*–ingredients–therapeutic genes–VMC” network.

A total of 202 ingredients of *S. miltiorrhiza* and 1179 targets influenced by it were identified using TCMSP and Swiss Target Prediction. Additionally, 1322 targets linked to VMC were obtained from GeneCards and OMIM. By intersecting the drug and disease targets, 257 therapeutic targets were identified (Figure 1A). The protein–protein interactions (PPI) of these therapeutic targets were analyzed using STRING, where different colored ellipses represent individual targets, and the connecting lines indicate interactions (Figure 1B). Using Cytoscape 3.10.3, 106 hub genes were identified based on three centrality metrics—Degree, Betweenness, and Closeness—retaining those ranked in the top 50% for each metric, with 3615 interactions found among these hub genes (Figure 1C). To elucidate the relationship between *S. miltiorrhiza* and VMC, we constructed a “*Salvia miltiorrhiza*–ingredients–therapeutic genes–VMC” network that systematically depicts how the herb components modulate therapeutic genes associated with VMC (Figure 1D). Ranking ingredients by the number of therapeutic genes they affect in the network revealed ten hub components: apigenin (MOL000008), luteolin (MOL000006), ursolic acid (MOL000511), oleic acid (MOL000675), 2-(4-hydroxy-3-methoxyphenyl)-5-(3-hydroxypropyl)-7-methoxy-3-benzofurancarboxaldehyde (MOL007050), dan-shexinkum D (MOL007093), danshenol A (MOL007082), 2-isopropyl-8-methylphenanthrene-3,4-dione (MOL007041), cryptotanshinone (MOL007088), and methylrosmarinate (MOL007116). Dan-shexinkum D, danshenol A, cryptotanshinone, and methylrosmarinate are identified as key ingredients due to their specificity to *S. miltiorrhiza* or their relatively high concentrations [20,21].

### 2.2. GO and KEGG Analysis of Therapeutic Targets

**Figure 2:**
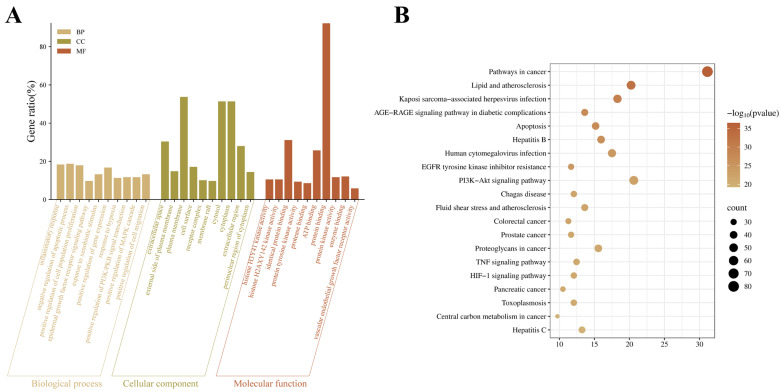
GO enrichment and KEGG pathway analysis of therapeutic genes. (**A**) Top 10 enriched terms for biological process (BP), cellular component (CC), and molecular function (MF) among therapeutic targets. (**B**) Top 20 KEGG pathway entries from the analysis.

Gene Ontology (GO) enrichment and KEGG pathway analyses of therapeutic targets were performed using DAVID. Only data with *p*-value < 0.01 and FDR < 0.01 were retained. According to the *p*-values ranked from small to large, GO analysis showed that the biological processes through which *S. miltiorrhiza* acts against VMC mainly involve inflammatory response, negative regulation of apoptotic process, and positive regulation of cell population proliferation. The relevant cellular components include extracellular space, external side of plasma membrane, and plasma membrane. The principal molecular functions comprise histone H3Y41 kinase activity, histone H2AXY 142 kinase activity, and identical protein binding (Figure 2A). KEGG enrichment highlighted Pathways in cancer, Lipid and atherosclerosis, and Kaposi sarcoma-associated herpesvirus infection, ranked by the number of enriched genes (Figure 2B).

### 2.3. Machine Learning-Guided Identification of Core Therapeutic Targets

**Figure 3:**
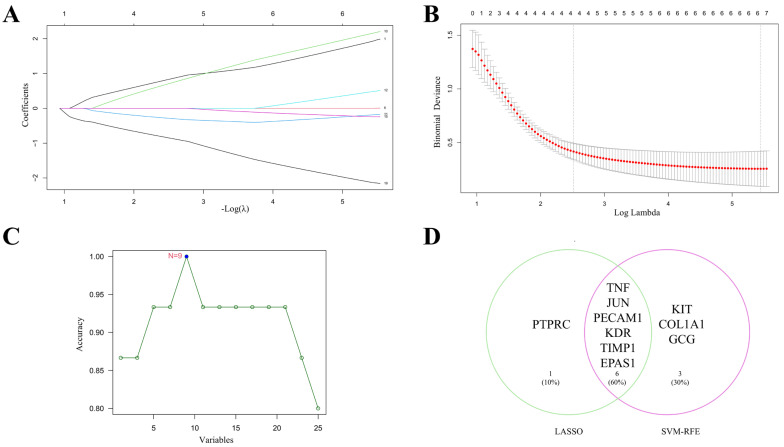
Machine learning identification of core therapeutic targets. (**A**,**B**) Results of Least Absolute Shrinkage and Selection Operator (LASSO) regression. The upper abscissa denotes the number of model genes at different λ values, and the optimal λ corresponds to 7 genes. (**C**) Support Vector Machine (SVM) results, which reached maximum accuracy with 9 genes. (**D**) Six genes were identified as core therapeutic targets of *S. miltiorrhiza* for VMC by intersecting the LASSO and SVM results.

Transcriptomic data for VMC (GSE183850) were retrieved from GEO, and differentially expressed genes were identified using thresholds of fold change >2 or <0.5 and *p*-value < 0.05. Intersecting these genes with the hub genes from Section 2.1 yielded 26 candidate genes. To identify core therapeutic targets among these 26 genes, we applied Least Absolute Shrinkage and Selection Operator (LASSO) regression and support vector machine (SVM) algorithms. LASSO selected 7 core targets; in the coefficient plot, different colored lines represent individual genes, and the “Coefficients” indicate each gene’s contribution to the effect of *S. miltiorrhiza* in VMC (Figure 3A). The upper abscissa displays the number of model genes for varying λ values, with the optimal λ corresponding to a model of 7 genes (Figure 3B). The SVM approach identified 9 core targets (Figure 3C). The intersection of LASSO and SVM results produced 6 core therapeutic targets: TNF, JUN, PECAM1, KDR, TIMP1, and EPAS1 (Figure 3D).

### 2.4. In Silico Docking of Key Ingredients to Core Therapeutic Targets

**Figure 4:**
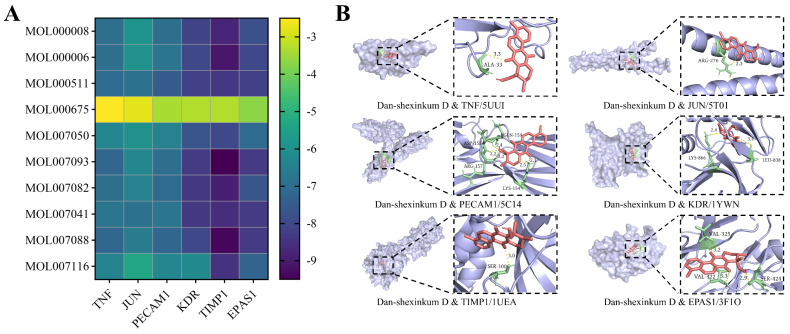
In silico docking poses and binding interactions of key ingredients with core therapeutic targets. (**A**) Heatmap of in silico docking between ten hub components of *S. miltiorrhiza* and core therapeutic targets, showing that dan-shexinkum D (MOL007093) had the lowest total binding energy. (**B**) Docking poses of dan-shexinkum D with six core therapeutic targets.

Ten hub components of *S. miltiorrhiza*, as identified in Section 2.1, were docked to six core therapeutic targets: TNF (PDB ID: 5UUI; *x* center = 45.30, *y* center = 52.93, *z* center = 13.77), JUN (PDB ID: 5T01; *x* center = −16.56, *y* center = 23.05, *z* center = 26.23), PECAM1 (PDB ID: 5C14; *x* center = 20.01, *y* center = 32.69, *z* center = −0.87), KDR (PDB ID: 1YWN; *x* center = 5.03, *y* center = 38.56, *z* center = 23.56), TIMP1 (PDB ID: 1UEA; *x* center = 60.04, *y* center = 61.00, *z* center = 38.77), and EPAS1 (PDB ID: 3F1O; *x* center = 11.33, *y* center = −52.20, *z* center = 16.07), utilizing AutoDock Vina 1.1.2. A ligand–receptor pair was considered to have a stable binding state when the binding energy was less than −5 kcal/mol. Except for oleic acid (MOL000675), all nine remaining ingredients showed stable binding to the core therapeutic targets, and dan-shexinkum D (MOL007093) had the lowest total binding energy among them (Figure 4A). Docking poses of dan-shexinkum D with the receptor proteins were visualized in PyMOL 3.0 (Figure 4B).

### 2.5. Molecular Dynamics Simulation of Dan-Shexinkum D and Core Therapeutic Targets

**Figure 5:**
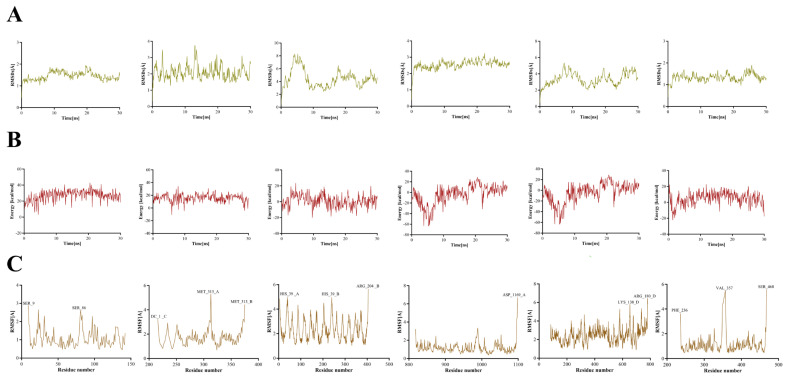
Molecular dynamics simulation of dan-shexinkum D with six core therapeutic targets. (**A**) Displays RMSD results for dan-shexinkum D interacting with TNF, JUN, PECAM1, KDR, TIMP1 and EPAS1. (**B**) Shows Bind Energy outcomes for dan-shexinkum D and TNF, JUN, PECAM1, KDR, TIMP1 and EPAS1. (**C**) Illustrates RMSF data for dan-shexinkum D TNF, JUN, PECAM1, KDR, TIMP1 and EPAS1.

To further evaluate the binding affinity of key *S. miltiorrhiza* components for core therapeutic targets, molecular dynamics simulations of dan-shexinkum D bound to each of six core therapeutic targets were conducted in YASARA 10.3.16. Complex stability was assessed by root-mean-square deviation (RMSD) and binding energy, and root-mean-square fluctuation (RMSF) was used to identify residues that acquired high flexibility upon binding. RMSD measures the deviation in the protein–ligand complex from a reference structure (for example, the initial crystal structure) during the simulation and thus serves as the primary indicator of equilibration and structural stability [22]. The RMSD profiles indicate that the dan-shexinkum D–target complexes remain relatively stable, with the dan-shexinkum D–TNF and dan-shexinkum D–EPAS1 complexes showing smaller RMSD fluctuations, which reflects a more stable interaction state for these two complexes (Figure 5A). System energy stability is another key criterion for assessing binding persistence, since large fluctuations can indicate potential conformational change. The relatively stable binding of dan-shexinkum D to the six target proteins are reflected by the low fluctuations in the complexes’ binding energy (Figure 5B). RMSF quantifies the amplitude of motion of each residue or atom about its mean position, with higher RMSF indicating greater local flexibility that is often associated with activity or binding [23]. Except for the N- and C-termini, residues at the dan-shexinkum D binding sites showed relatively low RMSF, and all residues corresponding to pronounced peaks are labeled (Figure 5C).

### 2.6. Meta-Analysis of Salvia miltiorrhiza Injection for Viral Myocarditis

**Figure 6:**
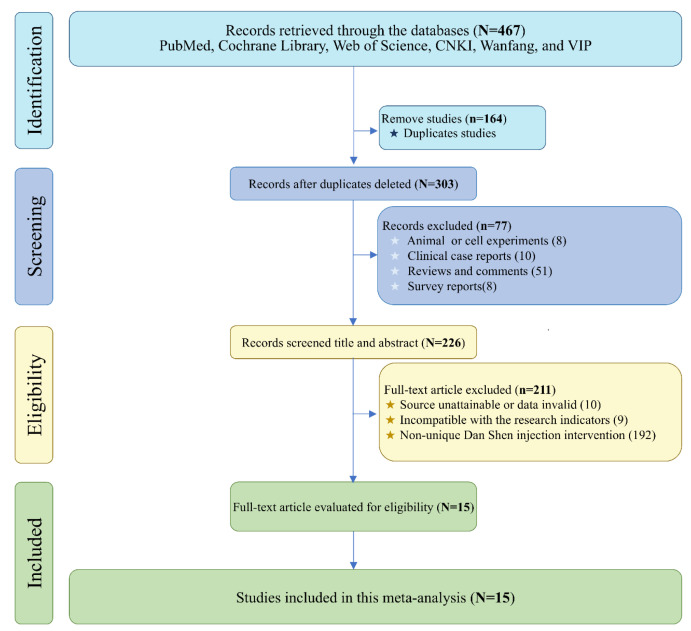
The relevant literature of *Salvia miltiorrhiza* Injection against viral myocarditis search flow chart.

A total of 467 publications were retrieved from PubMed, Web of Science, Cochrane Library, CNKI, Wanfang, and VIP using the primary search terms “*Salvia miltiorrhiza*”, “Danshen”, and “Viral Myocarditis”. After screening and eligibility assessment, 15 studies were included in this meta-analysis [24,25,26,27,28,29,30,31,32,33,34,35,36,37,38] (Figure 6 and Table 1).

**Figure 7:**
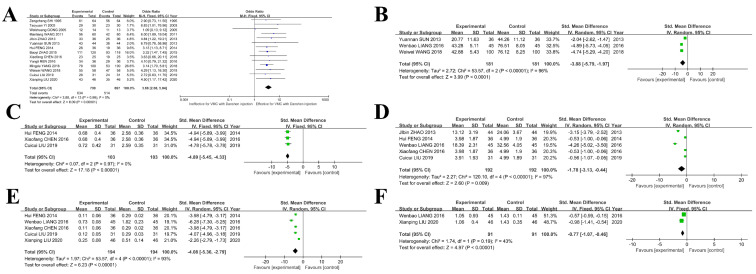
Meta-analysis results of *Salvia miltiorrhiza* Injection for viral myocarditis. (**A**) Forest plot depicting the treatment effectiveness of *Salvia miltiorrhiza* injection for VMC. (**B**) Impact on the TNF-α index during the treatment process with *S. miltiorrhiza* for VMC. (**C**) Effectiveness of the injection on hypersensitive C-reactive protein (hs-CRP) in VMC. (**D**) Changes in the CK-MB index. (**E**) Impact of Cardiac troponin I (cTnT) index. (**F**) Effect on Heat-fatty acid-binding protein (H-FABP) [24,25,26,27,28,29,30,31,32,33,34,35,36,37,38].

Fourteen studies documented the effectiveness of *S. miltiorrhiza* injection for treating VMC, and a meta-analysis of these studies demonstrated its efficacy [OR = 3.68, 95%CI (2.68~5.04)] (Figure 7A). Treatment effects were also reported as changes in VMC clinical indicators in some of the 15 included studies during therapy with *S. miltiorrhiza* injection. The meta-analysis found that the injection significantly reduced TNF-α [SMD = −3.88, 95%CI (−5.79~−1.97)] (Figure 7B), hypersensitive C-reactive protein [SMD = −4.89, 95%CI (−5.45~−4.33)] (Figure 7C), CK-MB [SMD = −1.78, 95%CI (−3.13~−0.44)] (Figure 7D), cardiac troponin I [SMD = −4.08, 95%CI (−5.36~−2.79)] (Figure 7E), and heart-type fatty acid-binding protein [SMD = −0.77, 95%CI (−1.07~−0.46)] (Figure 7F).

**Figure 8:**
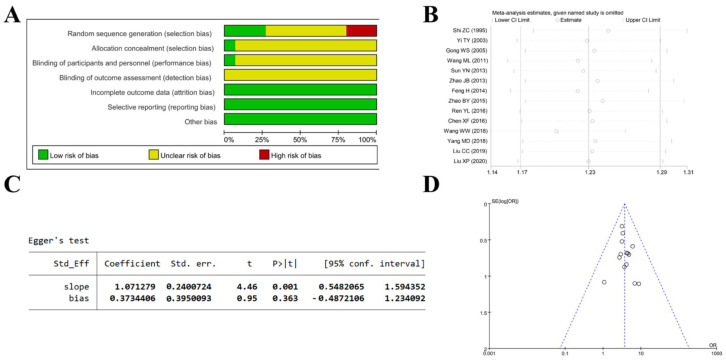
Quality assessment of included studies. (**A**) Risk-of-bias evaluation of the included studies using the seven Cochrane domains. (**B**) Sensitivity analysis of the included studies. (**C**) Assessment of publication bias using Egger’s test. (**D**) Funnel plot illustrating publication bias in the included studies [24,25,26,27,28,29,31,32,33,34,35,36,37,38].

Risk-of-bias assessment, sensitivity analysis, and publication-bias evaluation were performed to assess the overall quality of the included studies [39]. The risk-of-bias assessment indicates an overall unclear risk, particularly for random sequence generation and blinding (Figure 8A). Sensitivity analysis shows that effect estimates for all efficacy outcomes lie within the 95% CI, indicating consistency across studies and that the pooled results are still robust to exclusion of any single study (Figure 8B). Egger’s test yielded *p* = 0.363, and the funnel plot shows all points within the 95% confidence interval and roughly symmetric around the central line, indicating minimal publication bias (Figure 8C,D).

## 3. Discussion

VMC is an inflammatory, nonischemic injury of the myocardium triggered by viral infection, with a lack of pathogenesis-targeted therapies contributing to persistently poor clinical outcomes [40]. Current clinical care relies on multimodal supportive strategies, and *S. miltiorrhiza* injection is used as an adjunct with reported benefit [24,25,26,27,28,29,30,31,32,33,34,35,36,37,38]. However, its molecular pharmacology remains unclear, and no systematic review or meta-analysis has yet to be validated. To address this evidence gap, we applied multiple computational approaches to delineate likely molecular mechanisms and conducted a comprehensive meta-analysis to evaluate clinical effectiveness. Our computational mechanistic investigation and evidence-based synthesis provide a clinical and translational rationale for using *S. miltiorrhiza* and its injection to treat VMC.

Excessive inflammation, triggered by various factors beyond direct viral cytopathic effects, plays a crucial role in myocardial injury in VMC, leading to rapid clinical deterioration [41,42]. Our study provides both computational and clinical evidence that highlights the role of *S. miltiorrhiza* in reducing hyper-inflammatory responses, which is a key component of its therapeutic potential. We conducted function enrichment analyses (GO and KEGG) on identified 257 therapeutic targets through network pharmacology. The GO analysis identified the “inflammatory response” as the primary biological process. Concurrently, the PI3K-Akt, TNF, and HIF-1 signaling pathways, which are closely linked to the inflammatory response, were among the top 20 pathways in the KEGG results [43,44]. Through multi-computational approaches and transcriptomic data, we identified six core targets (*TNF*, *JUN*, *PECAM1*, *KDR* (*VEGFR2*), *TIMP1*, and *EPAS1* (*HIF-2α*)) closely associated with inflammation, vascular remodeling, and fibrosis suppression [45,46,47,48,49]. This suggests that *S. miltiorrhiza* likely coordinates an anti-inflammatory (*TNF/JUN/PECAM1*)—pro-resolving angiogenic (*KDR/EPAS1*)—anti-fibrotic (*TIMP1*) axis in VMC treatment. Furthermore, our meta-analysis reinforces the efficacy of the herb in attenuating inflammation in VMC, showing that adjunctive *S. miltiorrhiza* injection significantly lowers circulating levels of TNF-α and hypersensitive C-reactive protein (hs-CRP) in VMC patients. While the suppression of inflammatory cascades by *S. miltiorrhiza* has been reported in other conditions [50], our study is the first to elucidate its significance and potential molecular mechanisms in VMC, supported in part by clinical evidence.

In molecular docking, all four key ingredients (dan-shexinkum D, danshenol A, cryptotanshinone, and methylrosmarinate) demonstrated strong binding to the core therapeutic target. Among them, dan-shexinkum D exhibited the lowest total binding energy, and its stable binding was confirmed through molecular dynamics simulations. There are successful precedents for purifying *S. miltiorrhiza* components and developing them into drugs. Magnesium Lithospermate B, produced by complexing salvianolic acid B with magnesium ions, has been approved in China and is widely used to treat cardiovascular conditions such as coronary heart disease and angina pectoris [51]. Among the four key ingredients, cryptotanshinone demonstrates pharmacological effects in cardiovascular, cancer, and nervous system diseases, while danshenol A has been shown to inhibit inflammation and alleviate hypertension-induced cardiac remodeling [52,53,54]. However, research on dan-shexinkum D and methylrosmarinate remains limited. Our study offers insights into the pharmacological potential of these four components and advocates for further research into their mechanisms and structural modifications to advance their development as novel drugs for VMC and related diseases.

Current clinical practice for treating VMC with *S. miltiorrhiza* relies mainly on injections prepared from the single herb and on compound CHIs that include it as adjunct [55,56]. Despite multiple randomized controlled trials assessing the efficacy of *S. miltiorrhiza* injection for VMC, no dedicated systematic review or meta-analysis has synthesized these findings, which has limited its clinical adoption. Consequently, we performed meta-analysis revealing that this injection is efficacious in VMC treatment, leading to notable decreases in myocarditis-associated clinical indicators such as CK-MB, cTnT, and H-FABP [57,58]. However, the risk-of-bias assessment for the included studies was largely unclear, particularly for random sequence generation and blinding, which undermines the overall study quality. Future research should therefore prioritize rigorous randomized, controlled, double-blind clinical trials to strengthen the evidence base for this injection in VMC treatment.

Our study investigated potential mechanisms by which *S. miltiorrhiza* might combat VMC using various computational methods. However, these in silico predictions lack experimental validation and cannot replace such empirical evidence. Thus, the results should be considered preliminary, serving as a reference for identifying mechanisms and key components of the herb related to VMC, and guiding further translational and clinical research. Experimental validation is necessary, such as assessing mRNA or protein levels of key targets in in vivo or in vitro models treated with and without *S. miltiorrhiza*. Interactions among the core therapeutic targets *TNF*, *JUN*, *PECAM1*, *KDR*, *TIMP1*, and *EPAS1*, and among the PI3K–Akt, TNF, and HIF–1 signaling pathways, could be investigated by establishing VMC models with cardiotropic viruses such as CVB3 and then treating them with *S. miltiorrhiza* or its injection [59]. Similarly, further exploration of the pharmacological effects of key components is necessary. For instance, compounds such as dan-shexinkum D should be used in cell or animal models of VMC to examine their pharmacological potential and underlying mechanisms.

In conclusion, this study identified the key constituents and potential pharmacological mechanisms of *S. miltiorrhiza* against VMC using multiple computational approaches, established an evidence-based rationale for its injection, and provided a preliminary foundation for future clinical translation and application.

## 4. Materials and Methods

### 4.1. Acquisition of Drug, Disease and Therapeutic Targets Data

The components of *S. miltiorrhiza* and their corresponding targets were identified using TCMSP (https://go.drugbank.com/, accessed on 31 October 2025) with the search term “Danshen”, the Chinese name of *Salvia miltiorrhiza*. After downloading the SDF format of these ingredients from TCMSP, they were uploaded to Swiss Target Prediction (https://go.drugbank.com/, accessed on 31 October 2025) to determine the targets recorded in this database. The targets obtained from TCMSP were converted to standard gene names using Uniprot IDs from the Uniprot database (https://www.uniprot.org/, accessed on 31 October 2025). Targets related to VMC were sourced from GeneCards (https://www.genecards.org/, accessed on 31 October 2025) and Online Mendelian Inheritance in Man (https://www.omim.org/, accessed on 31 October 2025). The therapeutic targets of *S. miltiorrhiza* for VMC were determined by intersecting the drug and disease targets using Venny 2.1.0 (https://bioinfogp.cnb.csic.es/tools/venny/, accessed on 31 October 2025).

### 4.2. PPI Network Construction and Hub Targets Validation

Using STRING (https://cn.string-db.org/, accessed on 31 October 2025), we generated a protein–protein interaction (PPI) network by uploading the therapeutic targets identified in Section 4.1. From the PPI dataset, we selected hub targets using three centrality metrics—Degree, Betweenness, and Closeness—and retained genes ranked in the top 50% for each metric using Cytoscape 3.10.3 (Institute for Systems Biology, Seattle, WA, USA) [60]. The 50% threshold was selected to ensure a substantial pool of candidates for reliable machine-learning modeling without excessively strict filtering that could eliminate valuable features. To visualize potential links between *S. miltiorrhiza* and VMC, we then built a “Drug–Ingredients–Therapeutic genes–Disease” network in Cytoscape 3.10.3. Hub components were defined by the number of therapeutic genes they affected within the network. Key ingredients were then selected from those hub components based on their specificity or high content in *S. miltiorrhiza*, as reported in the previous literature.

### 4.3. Functional Enrichment Analysis of Therapeutic Genes

Functional enrichment analysis (GO and KEGG) was performed on the therapeutic targets dataset using the Database for Annotation, Visualization, and Integrated Discovery (DAVID; https://davidbioinformatics.nih.gov/, accessed on 31 October 2025). GO analysis encompassed biological process (BP), cellular component (CC), and molecular function (MF). The top 10 entries from each GO category and the top 20 KEGG pathways were exported for visualization with Bioinformatics (https://www.bioinformatics.com.cn/, accessed on 31 October 2025).

### 4.4. Machine Learning for Identification of Core Therapeutic Targets

Transcriptomic data for VMC were obtained from GEO (https://www.ncbi.nlm.nih.gov/geo/, accessed on 31 October 2025). Significant differential genes were defined as those with fold change >2 or <0.5 and *p*-value < 0.05. Reliable therapeutic targets were identified as the intersection of hub genes and these significant differential genes. From these targets, two machine-learning algorithms—Least Absolute Shrinkage and Selection Operator (LASSO) regression and Support Vector Machine (SVM)—were applied to identify core therapeutic targets. LASSO regression was run with the R 4.4.3 package glmnet using standardize = TRUE, alpha = 1, family = “binomial”, and ten-fold cross-validation was used and that the final penalty corresponds to the “lambda.min” criterion. SVM was implemented with the linear kernel (svmLinear), the default cost parameter exactly as implemented by caret’s svmLinear method (i.e., no user tuning), Z-score standardization (centering and scaling via preProcess) performed before training, and the stopping rule defined by sizes = c(seq(1, 23, by = 2)).

### 4.5. In Silico Docking of Key Components to Core Therapeutic Targets

Hub components of *S. miltiorrhiza* were identified by the “Drug–Ingredients–Therapeutic targets–Disease” network in Section 4.2 according to the number of influenced targets (quasi “Degree”). SDF files of the hub components were retrieved from PubChem (https://pubchem.ncbi.nlm.nih.gov, accessed on 31 October 2025), energy-minimized to their lowest-energy conformers with Chem3D 22.0.0 (CambridgeSoft, Cambridge, MA, USA), and exported in MOL2 format. PDB files of the core therapeutic targets were downloaded from RCSB (https://www.rcsb.org/, accessed on 31 October 2025) and had water molecules and organic ligands removed using PyMOL 3.0 (Schrödinger, South San Francisco, CA, USA). In silico docking between the hub components and core therapeutic targets was performed with AutoDock Vina 1.1.2 (Scripps Research, La Jolla, CA, USA) using AutoDockTools 1.5.7 (Scripps Research, La Jolla, CA, USA), with the docking grid box spacing set to 1 Å. Docking results were visualized in PyMOL 3.0.

### 4.6. Molecular Dynamics Simulation of Key Components with Core Therapeutic Targets

The receptor protein and the ligand (dan-shexinkum D) were prepared using YASARA 10.3.16 (YASARA Biosciences GmbH, Vienna, Austria). This involved removing water molecules, ions, and any co-crystallized ligands, adding hydrogen atoms, and assigning protonation states at pH 7.4. Molecular docking was subsequently performed, and the complex exhibiting the most favorable binding energy and stereo-chemically reasonable geometry was selected for molecular dynamics simulation. The selected complex was parameterized using the AMBER force field and solvated in a cubic box of explicit solvent environment. A cubic simulation box was built using periodic boundary conditions in “around-all-atoms” mode with a 5 Å buffer (α = β = γ = 90°), and water molecules and ions were added using the software defaults. The root-mean-square deviation (RMSD), binding energy, and root-mean-square fluctuation (RMSF) were recorded throughout the 30 ns (30,000 ps) simulation. The resulting trajectories were analyzed, and the data were visualized as line plots using GraphPad Prism 10.4.2 (GraphPad Software, San Diego, CA, USA).

### 4.7. Meta-Analysis of Salvia miltiorrhiza Injection for Viral Myocarditis

We searched PubMed, Web of Science, the Cochrane Library, and three Chinese databases (CNKI: https://www.cnki.net/, accessed on 31 October 2025; Wanfang: https://www.wanfangdata.com.cn/, accessed on 31 October 2025; VIP: https://qikan.cqvip.com/, accessed on 31 October 2025) for studies of *S. miltiorrhiza* injection for VMC from inception through November 2025. Drug search terms included “*Salvia miltiorrhiza*” and “Danshen,” and disease terms included “myocarditis” and “viral myocarditis”. We used a combined subject heading and keyword strategy with no language or geographic restrictions. For example, PubMed searches combined the MeSH terms “*Salvia miltiorrhiza*”[Mesh] and “Myocarditis”[Mesh] with free text words such as “Danshen”, “Dan Shen”, “Chinese *Salvia*”, “*Salvias*, Chinese”, “Tan Seng”, and “viral myocarditis”.

The retrieved literature was screened based on the inclusion and exclusion criteria of this study. Studies were included if they met all of the following: (1) the population comprised patients diagnosed with VMC according to established guidelines; (2) the design was a randomized controlled trial (RCT); (3) the only difference in intervention between comparison groups was administration of *S. miltiorrhiza* injection from a single-herb source; and (4) the study reported treatment outcomes or relevant clinical indicators of VMC. Studies were excluded for any of the following: (1) noncontrolled designs, including reviews, retrospective analyses, and animal studies; (2) sources that could not be obtained; or (3) invalid original data or duplicate publications.

The risk-of-bias evaluation of the included studies was assessed using seven domains recommended by Cochrane. Key information, including group sizes (experiment and control) and outcome values, were extracted from the studies selected for the meta-analysis. The meta-analysis was performed using RevMan 5.4.1 (Epic Gecko Ltd., Bergen, Norway). Heterogeneity was determined using Cochrane’s *Q* test and the *I*^2^ statistic. A random-effects model was applied when there was significant heterogeneity in the data (*I*^2^ > 50% or *p* < 0.1), while a fixed-effects model was used for low heterogeneity (*I*^2^ ≤ 50% and *p* ≥ 0.1). Sensitivity analyses and evaluation of publication bias were conducted using Stata 18 (StataCorp LLC, College Station, TX, USA). Publication bias was also assessed visually with funnel plots in RevMan 5.4.1.

## 5. Conclusions

The core therapeutic targets of *S. miltiorrhiza* for VMC include *TNF*, *JUN*, *PECAM1*, *KDR*, *TIMP1*, and *EPAS1*, focusing on anti-inflammatory effects, vascular remodeling, and fibrosis suppression. Dan–shexinkum D, danshenol A, cryptotanshinone and methylrosmarinate were identified as key ingredients, with dan–shexinkum D demonstrating particularly promising results. When used as an adjunct therapy, *S. miltiorrhiza* injection effectively improves outcome and significantly reduces levels of TNF-α, hs-CRP, CK-MB, cTnT, and H-FABP.

## Figures and Tables

**Table 1:** Characteristics of included studies.

First Author and Publication Year	Number of Participants; Danshen and Control	Intervention		Frequency and Treatment Duration (d)	Jadad Score	Research Indicators
		Control Group	Danshen Group			
Liu XP 2020 [24]	46 & 46	Conventional treatment with levocarnitine (50 g/L, IV)	The control regimen plus *Salvia miltiorrhiza* injection (50 g/L, IV)	1 treatment per day for 2 weeks	1	①⑤⑥
Liu CC 2019 [25]	31 & 31	Conventional treatment plus vitamins C, E, and coenzyme Q10	The control regimen plus *Salvia miltiorrhiza* injection (20 mL in 250 mL of 5% glucose, IV)	1 treatment per day for 20 days	2	①③④⑤
Wang WW 2018 [26]	100 & 100	Conventional treatment plus fructose-1,6-diphosphate (FDP)	The control regimen plus *Salvia miltiorrhiza* injection (IV)	—	2	①②
Yang MD 2018 [27]	58 & 58	Conventional treatment plus fruc-tose-1,6-diphosphate (FDP)	The control regimen plus *Salvia miltiorrhiza* injection (5~15 mL in 5% glucose, IV)	1 treatment per day for 2 weeks	1	①
Ren YL 2016 [28]	25 & 25	Conventional treatment plus fruc-tose-1,6-diphosphate (FDP)	The control regimen plus *Salvia miltiorrhiza* injection (5~15 mL in 5% glucose, IV)	1 treatment per day for 2 weeks	3	①
Chen XF 2016 [29]	36 & 36	Conventional treatment plus fruc-tose-1,6-diphosphate (FDP)	The control regimen plus *Salvia miltiorrhiza* injection (5~15 mL in 5% glucose, IV)	1 treatment per day for 2 weeks	2	①③④
Liang WB 2016 [30]	45 & 45	Conventional treatment plus fruc-tose-1,6-diphosphate (FDP)	The control regimen plus *Salvia miltiorrhiza* injection (5~15 mL in 5% glucose, IV)	1 treatment per day for 2 weeks	2	②④⑤⑥
Zhao BY 2015 [31]	120 & 118	Fructose diphosphate sodium (100 mg/kg, IV)	The control regimen plus *Salvia miltiorrhiza* injection (0.5~1 mL/kg in 100~150 mL 5% glucose, IV)	1 treatment per day for 20 days	3	①
Feng H 2014 [32]	36 & 36	Conventional treatment plus fruc-tose-1,6-diphosphate (FDP)	The control regimen plus *Salvia miltiorrhiza* injection (5~15 mL in 5% glucose, IV)	1 treatment per day for 2 weeks	2	①③④⑤
Sun YN 2013 [33]	36 & 36	Conventional treatment plus vitamins C, E, and coenzyme Q10	The control regimen plus *Salvia miltiorrhiza* injection (20 mL in 250 mL of 5% glucose, IV)	1 treatment per day for 20 days	3	①②
Zhao JB 2013 [34]	44 & 44	Intravenous infusion of ribavirin (10–15 mg/kg), fructose-1,6-diphosphate (100–200 mg/kg), and vitamin C (100–200 mg/kg).	The control regimen plus *Salvia miltiorrhiza* injection (0.5 mL/kg in 100~150 mL 5% glucose, IV)	1 treatment per day for 2 weeks	3	①④
Wang ML 2011 [35]	44 & 44	Conventional treatment plus taurine (2 g three times daily)	The control regimen plus *Salvia miltiorrhiza* injection (40 mL in 100~200 mL 5% glucose, IV)	1 treatment per day for 4 weeks	2	①
Gong WS 2005 [36]	14 & 13	Conventional treatment plus fruc-tose-1,6-diphosphate (200 mg/kg)	The control regimen plus *Salvia miltiorrhiza* injection (0.2 mL/kg in 100 mL 10% glucose, IV)	Once-daily administration for two 2-week courses, separated by a 2-day interval.	2	①
Yi TY 2003 [37]	30 & 30	Conventional treatment plus vitamin C (100 mg/kg in 100 mL 5~10% glucose, IV).	The control regimen plus *Salvia miltiorrhiza* injection (0.5 mL/kg in 100 mL 5~10% glucose, IV)	1 treatment per day for 2 weeks	2	①
Shi ZC 1995 [38]	64 & 64	Conventional treatment	Conventional treatment plus *Salvia miltiorrhiza* injection (30 mL in 250~500 mL 10% glucose, IV)	Once-daily administration for two 2-week courses, separated by a 5-day interval.	2	①

Note: Conventional treatment: antiviral agents, bed rest, immunomodulation, glucocorticoids (if necessary) and symptomatic treatments; Research Indicators: ① Treatment effect; ② TNF-α; ③ hypersensitive C-reactive protein (hs-CRP); ④ CK-MB; ⑤ Cardiac troponin I (cTnT); ⑥ Heat-fatty acid-binding protein (H-FABP).

## Data Availability

The original contributions presented in this study are included in the article. Further inquiries can be directed to the corresponding author.
